# Oral health knowledge, attitudes, and practices and associated factors among non-dental healthcare professionals in South China: a cross-sectional study

**DOI:** 10.3389/fpubh.2026.1811364

**Published:** 2026-04-30

**Authors:** Fanglin Xiao, Dandan Yu, Yingsi Huang, Ting Zhang, Hongkun He, Wei Luo, Lanfeng Ye, Wenguang Qin

**Affiliations:** Department of Periodontology, School and Hospital of Stomatology, Guangdong Engineering Research Center of Oral Restoration and Reconstruction, Guangzhou Medical University, Guangzhou, China

**Keywords:** attitude, clinical behavior translation, health education, IKAP, knowledge, practice

## Abstract

**Background:**

Non-dental healthcare professionals (NDHPs) play an important role in oral health promotion; however, evidence on their oral health knowledge, attitudes, and practices (KAP) remains limited. This study aimed to assess oral health knowledge, attitudes, and practices (KAP) among NDHPs in South China and to identify key factors associated with their overall KAP level.

**Methods:**

A cross-sectional survey was conducted between October 26 and December 25, 2024, among 809 NDHPs from multiple healthcare institutions in South China. The questionnaire assessed oral health knowledge, attitudes, practices, and related training needs. The Kruskal–Wallis H test was used to compare KAP scores across professions; Spearman correlation analysis examined associations among KAP domains; and ordinal logistic regression identified factors associated with overall KAP level.

**Results:**

Overall, NDHPs demonstrated a pattern of positive attitudes but limited knowledge and suboptimal practices. The overall correct rate for oral health knowledge was 25.94%, with no significant differences across professions (H = 1.041, *p* = 0.594). Traditional media (books/journals/newspapers) was the most common information source (56.9%), and 41.16% of participants had received systematic oral health education. Although 85.4% considered oral health “very important,” notable misconceptions remained. Nearly all participants reported brushing daily (97.0%), yet only 36.1% met the recommended brushing duration; use of fluoridated toothpaste (44.1%) and dental floss (<50%) was low. Attitudes were positively correlated with both knowledge and practices, whereas knowledge was not significantly correlated with practices. In regression analyses, higher educational attainment (OR = 3.29, *p* = 0.007), prior oral health education (OR = 3.62, *p* < 0.001), and age 45–54 years (OR = 2.04, *p* = 0.001) were associated with higher KAP levels, while being married was associated with lower scores (OR = 0.24, *p* = 0.025).

**Conclusion:**

NDHPs in South China exhibited generally positive attitudes toward oral health, but limited knowledge reserves and incomplete translation of knowledge into healthy practices. Attitude appears to be a key intermediary between knowledge and practice. Age, education, marital status, and prior oral health education were major determinants of oral health -related KAP in this population. These findings may inform efforts to strengthen oral health education within medical curricula and develop stratified, tailored training programs for NDHPs.

## 1 Introduction

Oral health is an essential component of overall health. Oral diseases are among the most prevalent non-communicable diseases worldwide, affecting more than 3.5 billion people (1). Their consequences extend beyond local problems such as tooth defects, tooth loss, and maxillofacial infections (2) and are closely linked to systemic conditions including diabetes, cardiovascular disease, and respiratory disease (3). Moreover, the costs of treatment impose a long-term socioeconomic burden (4).

As one of the earliest and most frequently patient-facing groups in healthcare settings, NDHPs’ oral health knowledge, attitudes, and practices can directly influence the quality and effectiveness of patient education (5). While many studies focus on dental professionals, evidence regarding NDHPs is limited, which may contribute to a disconnect between oral disease prevention and broader systemic health management in routine clinical practice (6–8).

Prior evidence suggests that NDHPs generally perform worse than dental professionals in key knowledge and practice domains such as professional dental cleaning (PDC) (9). Inadequate oral health knowledge among specific NDHP subgroups (e.g., nursing staff and obstetric providers) may adversely affect patient outcomes-for example, gaps in nurses’ oral care knowledge and training may limit their ability to guide patients (10), and limited awareness among obstetric providers may hinder identification and management of pregnancy risks associated with periodontal disease (11, 12). Therefore, the widespread oral health knowledge gap among NDHPs represents a potentially important but underrecognized public health risk.

The Information-Knowledge-Attitude-Practice (IKAP) model extends the classic KAP framework by emphasizing a stepwise process from information acquisition to knowledge internalization, attitude/belief formation, and ultimately behavior change, forming an actionable and evaluable closed loop that is particularly suitable for the design and evaluation of health promotion programs (13). In oral health research, the IKAP model has been applied successfully in populations such as older adults with periodontitis (14) and school-aged children and their caregivers (15–17), leading to significant improvements in knowledge, attitudes, and practices. Although these target groups differ from NDHPs, the core mechanism and layered evaluation logic of IKAP provide a useful theoretical lens for understanding NDHPs’ oral health-related knowledge, attitudes, practices, and behavioral drivers.

Systematic research on oral health -related KAP among frontline NDHPs remains insufficient (9, 10, 15), especially in South China, where healthcare resources are unevenly distributed and the region faces rapid population aging and a substantial chronic disease burden (18, 19). Whether NDHPs receive systematic oral health training affects not only their own health management but also their ability to provide early guidance and prevention counseling to patients, potentially constraining improvements in regional oral health (9).

Guided by the IKAP model, this study conducted a cross-sectional survey among NDHPs in South China to comprehensively assess oral health knowledge, attitudes, and practices and to identify key associated factors, providing evidence to support the development of tailored oral health promotion strategies for this population.

## 2 Materials and methods

### 2.1 Study design and participants

This study aimed to assess oral health-related KAP among NDHPs in South China and to identify factors associated with overall KAP level. We conducted a cross-sectional survey between October 26 and December 25, 2024, among NDHPs working in multiple healthcare institutions in South China Inclusion criteria were: (1) non-dental profession with a valid medical practice license; (2) at least 1 year of clinical experience; and (3) provision of informed consent and willingness to participate. Exclusion criteria were: (1) a history of hereditary or neoplastic oral diseases; or (2) inability to complete the survey. The study was approved by the Ethics Committee of the Affiliated Stomatology Hospital of Guangzhou Medical University (Approval No. LCYJ2024039). The questionnaire contained 25 items. Based on a sample size of 5–10 participants per item and an additional 20% allowance for invalid responses, the required sample size was estimated at 145–300; a total of 809 participants were ultimately included (20).

### 2.2 Instrument

A 25-item questionnaire (total score: 45) was developed based on the IKAP framework, with reference to the World Health Organization oral health reports, findings from the Fourth National Oral Health Epidemiological Survey of China, and relevant domestic and international literature (7, 8, 10, 13). Six experts in clinical medicine, nursing, and dentistry evaluated content validity, yielding a scale-level content validity index (S-CVI) of 0.987. A pilot survey of 50 NDHPs was conducted to assess clarity and avoid ambiguous wording. The attitude domain consisted of Likert-type items and was therefore considered suitable for internal consistency assessment. The Cronbach’s alpha coefficient for this domain was 0.709, indicating acceptable reliability (21). In contrast, the knowledge and behavior domains were primarily designed to assess participants’ mastery and implementation of multiple distinct oral health knowledge points and behavioral practices. Because these items were not intended to represent a highly homogeneous construct, internal consistency was not treated as the primary reliability evidence for these two domains; instead, greater emphasis was placed on content validity and the appropriateness of item design (22). The final questionnaire comprised five parts: (1) sociodemographic characteristics (sex, age, education, years of work, professional title, birthplace, marital status, profession, prior oral health education, and whether seeking care promptly when experiencing oral discomfort); (2) knowledge (10 items on periodontal risk factors, interdental cleaning tools, primary causes of tooth loss, etc.; 1 point per correct response; 8–10 points indicating high knowledge (23)); (3) attitudes (5 items scored on a 5-point Likert scale from 1 = strongly disagree to 5 = strongly agree; 20–25 points indicating positive attitudes (24)); (4) practices (10 items on brushing frequency and duration, and regular periodontal maintenance; 1 point per correct response; 8–10 points indicating high practice (23)); and (5) information sources and training needs.

### 2.3 Data collection

Senior nurses and the research team served as survey administrators. Administrators received standardized training before the survey to clarify study objectives and eligibility criteria. An electronic questionnaire was distributed via WeChat. Each IP address or WeChat account could submit the questionnaire only once, and submission was permitted only after all items were completed to minimize missing data. Questionnaires completed within 90 s, with incomplete/erroneous information, or with highly repetitive response patterns were considered invalid. Two researchers independently verified the dataset prior to statistical analysis.

### 2.4 Statistical analysis

Statistical analyses were performed using SPSS version 25.0. Categorical variables are presented as frequency and percentage [*n* (%)], and group differences were assessed using the chi-square test. Normality was evaluated using the Shapiro–Wilk test; *p* < 0.05 indicated non-normal distribution. For non-normally distributed continuous variables, nonparametric tests (Kruskal–Wallis H test or Mann–Whitney U test) were used. Associations among variables were examined using Spearman correlation analysis. Ordinal logistic regression was conducted to identify factors associated with overall oral health KAP level, reporting odds ratios (ORs) and 95% confidence intervals (CIs). A two-sided *p* < 0.05 was considered statistically significant.

## 3 Results

### 3.1 Participant characteristics

A total of 809 valid questionnaires were collected, including 227 clinicians, 272 nurses, and 310 participants from other medical disciplines (e.g., pharmacy, imaging, laboratory medicine, rehabilitation). Overall, 48.08% were male and 51.92% were female. Most participants held an intermediate or senior professional title (96.29%), and 58.84% reported no prior systematic oral health education. Further details are presented in Table 1.

**Table 1:** Demographic characteristics of non-dental healthcare professionals in South China (*N* = 809).

Category	Level	*n* (%)
1. Sex	Female	389 (48.08)
	Male	420 (51.92)
2. Age (years)	18–34	78 (9.64)
	35–44	347 (42.89)
	45–54	244 (30.16)
	≥55	140 (17.31)
3. Education	Bachelor’s degree or below	677 (83.68)
	Master’s degree or above	132 (16.32)
4. Years of work	1–5	24 (2.97)
	6–10	247 (30.53)
	11–15	99 (12.24)
	≥16	439 (54.26)
5. Professional title	Junior	30 (3.71)
	Intermediate	347 (42.89)
	Senior	432 (53.40)
6. Birthplace	Rural	106 (13.10)
	Urban	703 (86.90)
7. Marital status	Married	782 (96.66)
	Unmarried	27 (3.34)
8. Profession	Clinical medicine	227 (28.06)
	Nursing	272 (33.62)
	Other medical disciplines	310 (38.31)
9. Prior oral health education	Yes	333 (41.16)
	No	476 (58.83)
10. Seek care promptly when experiencing oral discomfort	Yes	399 (49.32)
	No	410 (50.68)

### 3.2 KAP scores by profession

Kruskal–Wallis H tests were used to compare KAP scores across professions (Table 2). Attitude, practice, and overall KAP scores differed significantly among the three groups (H = 13.534, *p* = 0.001; H = 6.795, *p* = 0.033; and H = 15.189, *p* = 0.001, respectively), with nurses showing the highest overall scores. Knowledge scores did not differ significantly (H = 1.041, *p* = 0.594).

**Table 2:** Comparison of knowledge, attitude, practice, and overall KAP scores across professions [median (P25, P75)].

Dimension	Clinical medicine (*N* = 227)	Nursing (*N* = 272)	Other medical disciplines (*N* = 310)	*H*	*p*
Knowledge	2 (1,3.5)	2 (2,4)	2 (2,4)	1.041	0.594
Attitude	18 (16,21)	19 (16,21)	18 (16,20)	13.534	0.001**
Behavior	6 (5,7)	6 (5,7)	6 (5,7)	6.795	0.033*
KAP	27 (23,29)	28 (24,31)	26 (24,29)	15.189	0.001**

*p < 0.05; **p < 0.01.

### 3.3 Oral health knowledge

**Figure 1:**
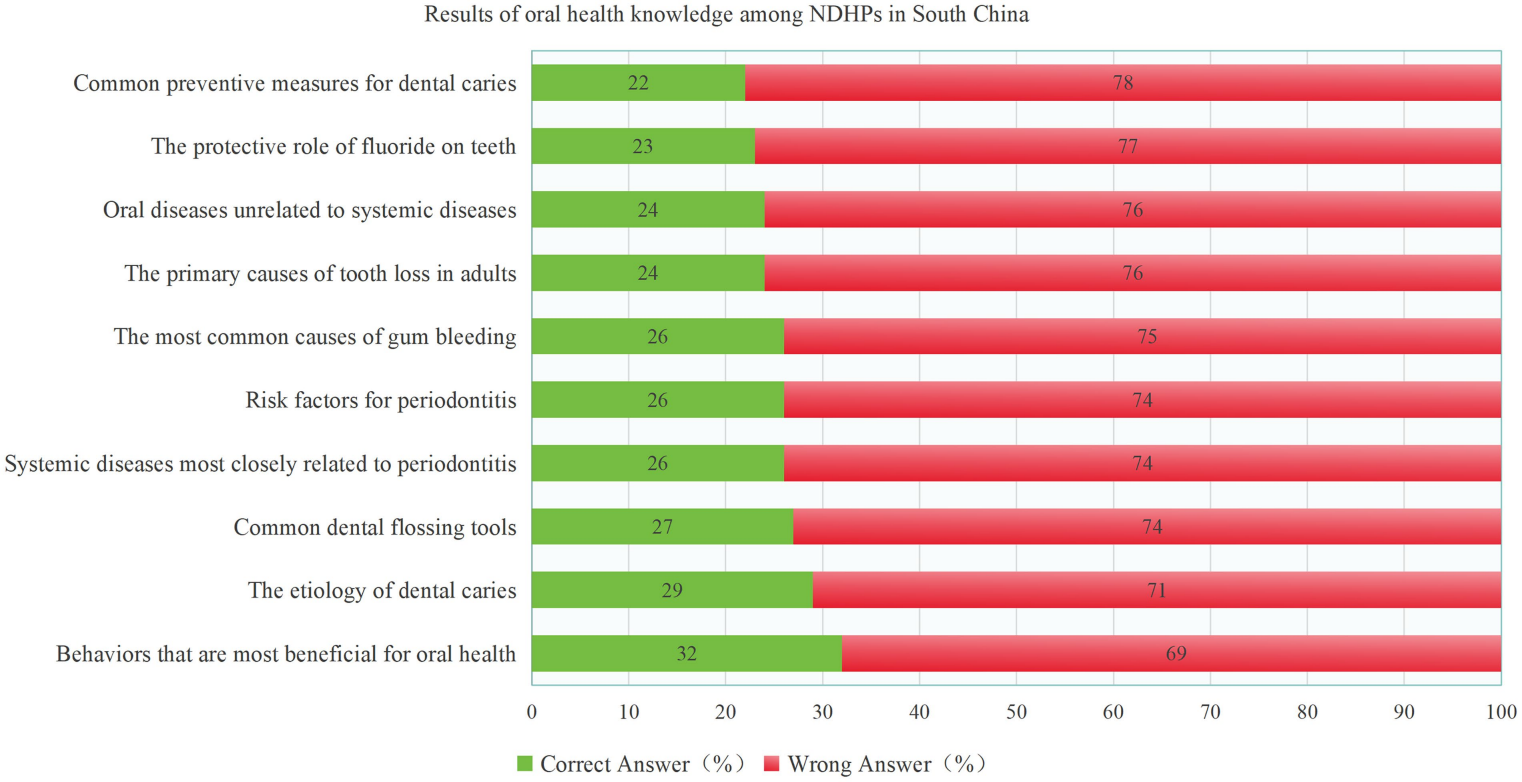
Results of oral health knowledge among NDHPs in South China.

Overall, nurses and participants from other medical disciplines showed slightly better performance than clinicians on specific knowledge items; however, the overall knowledge correct rate was low (25.94%). Across professions, 29.8% of nurses correctly recognized interdental brushes and dental floss as common interdental cleaning tools, compared with 29.5% of clinicians and 21.6% of other disciplines (*p* < 0.05). For the item on the most common cause of gingival bleeding, participants from other medical disciplines had the highest correct rate (*p* < 0.05). Notably, 39.0% of nurses identified the combination of regular toothbrushing, using dental floss to clean interdental spaces, and regular dental visits as the most beneficial behavior for oral health (Table 3). In addition, 77.1% of participants were unaware of the protective role of fluoride, and 77.6% were unfamiliar with common caries prevention measures such as fluoride use (Figure 1). Further subgroup comparison by prior oral health education showed that participants who had received oral health education had higher knowledge scores than those who had not, and the difference was statistically significant [3.00 (2.00, 4.00) vs. 2.00 (1.00, 3.00), Mann–Whitney U = 69540.500, Z = −3.037, *p* = 0.002].

**Table 3:** Oral health knowledge results.

Knowledge item	Correct response				*p*
	Clinical medicine (*N* = 227), *n* (%)	Nursing (*N* = 272), *n* (%)	Other medical disciplines (*N* = 310), *n* (%)	Total (*N* = 809), *n* (%)	
1. Which of the following is NOT a common interdental cleaning tool?	67 (29.5)	81 (29.8)	67 (21.6)	215 (26.6)	0.042
2. Which of the following is NOT a risk factor for periodontitis?	63 (27.8)	69 (25.4)	78 (25.2)	210 (26.0)	0.722
3. What is currently the primary cause of tooth loss in adults?	52 (22.5)	71 (26.1)	76 (24.5)	198 (24.5)	0.642
4. Which of the following diseases do you believe is most closely related to periodontal disease?	65 (28.6)	74 (27.2)	75 (24.2)	214 (26.5)	0.485
5. Which of the following is the most common cause of bleeding gums?	44 (19.4)	73 (26.8)	90 (29.0)	207 (25.6)	0.034
6. Dental caries is a common oral disease. Which of the following is NOT a cause of dental caries?	70 (30.8)	82 (30.1)	86 (27.7)	238 (29.4)	0.701
7. Which of the following is NOT a commonly used preventive measure for dental caries?	49 (21.6)	61 (22.4)	71 (22.9)	181 (22.4)	0.936
8. Which of the following behaviors is most beneficial for oral health?	61 (26.9)	106 (39.0)	88 (28.4)	255 (31.5)	0.005
9. What is the primary protective effect of fluoride on teeth?	43 (18.9)	59 (21.7)	83 (26.8)	185 (22.9)	0.087
10. Which of the following oral diseases is unrelated to systemic diseases?	62 (27.3)	61 (22.4)	71 (22.9)	194 (24.0)	0.379

### 3.4 Oral health attitudes

Most participants expressed positive attitudes toward oral health and believed that oral health is important in daily life. When experiencing gingival bleeding, tooth pain, or other symptoms, 85.4% reported that they would seek medical care promptly. However, misconceptions persisted; for example, 39.8% agreed to some extent that tooth quality is largely innate and is not strongly related to personal oral care (Table 4).

**Table 4:** Oral health attitude results.

Attitude item	Strongly disagree, *n* (%)	Disagree, *n* (%)	Neutral, *n* (%)	Agree, *n* (%)	Strongly agree, *n* (%)
1. Oral health care is important for people’s lives.	21 (2.6)	43 (5.3)	151 (18.7)	315 (38.9)	279 (34.5)
2. Tooth quality is innate and is not strongly related to personal oral care.	168 (20.8)	154 (19.0)	185 (22.9)	154 (19.0)	148 (18.3)
3. If there are health education lectures on oral care, I would be happy to attend.	40 (4.9)	99 (12.2)	170 (21.0)	231 (28.6)	269 (33.3)
4. If I experience symptoms such as bleeding gums or toothache, I will seek medical attention promptly.	38 (4.7)	80 (9.9)	185 (22.9)	238 (29.4)	268 (33.1)
5. Regular dental checkups are essential.	38 (4.7)	93 (11.5)	180 (22.2)	223 (27.6)	275 (34.0)

### 3.5 Oral health practices

For brushing duration and toothbrush selection, participants from other medical disciplines performed better than clinicians and nurses (*p* < 0.05). Significant differences were also observed in toothpaste type and dietary habits (*p* < 0.05). Nurses had the highest reported use of fluoridated toothpaste (44.1%), whereas participants from other disciplines reported a higher frequency of unfavorable dietary habits. Only 19.0% reported rinsing after meals (Table 5).

**Table 5:** Oral health practices results.

Practices item	Recommended/Correct practice				*p*
	Clinical medicine (*N* = 227), *n* (%)	Nursing (*N* = 272), *n* (%)	Other medical disciplines (*N* = 310), *n* (%)	Total (*N* = 809), *n* (%)	
1. How often do you brush your teeth each day?	220 (96.9)	262 (96.3)	303 (97.7)	785 (97.0)	0.598
2. How long do you brush your teeth each time?	68 (30.0)	86 (31.6)	138 (44.5)	292 (36.1)	0.000
3. How do you brush your teeth each time?	149 (65.6)	170 (62.5)	191 (61.6)	510 (63.0)	0.618
4. What type of toothbrush do you use when brushing your teeth?	172 (75.8)	233 (85.7)	250 (80.6)	655 (81.0)	0.019
5. How often do you replace your toothbrush?	82 (36.1)	119 (43.8)	123 (39.7)	324 (40.0)	0.220
6. Which method do you use to clean your teeth?	185 (81.5)	218 (80.1)	249 (80.3)	652 (80.6)	0.919
7. Do you rinse your mouth after meals?	43 (18.9)	51 (18.8)	60 (19.4)	154 (19.0)	0.982
8. Is the toothpaste you are currently using fluoride toothpaste?	78 (34.4)	120 (44.1)	74 (23.9)	272 (33.6)	0.000
9. How often do you typically consume sugary snacks, sweetened beverages, milk tea, tea, or coffee?	118 (52.0)	136 (50.0)	111 (35.8)	365 (45.1)	0.000
10. How often do you undergo dental check-ups and maintenance?	195 (85.9)	234 (86.0)	267 (86.0)	696 (86.0)	0.997

### 3.6 Oral health information sources and training needs

**Figure 2:**
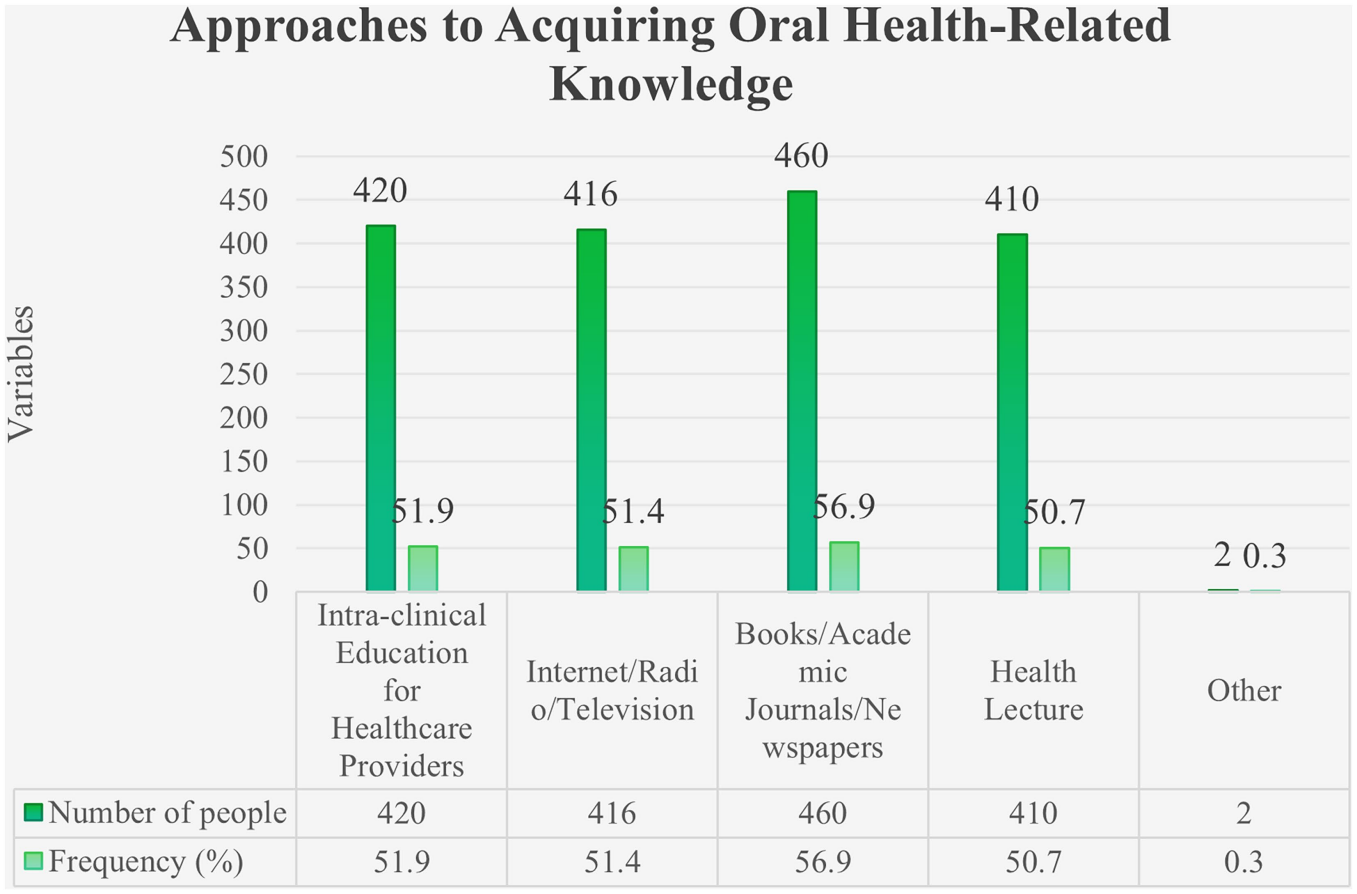
Approaches to acquiring oral health-related knowledge.

**Figure 3:**
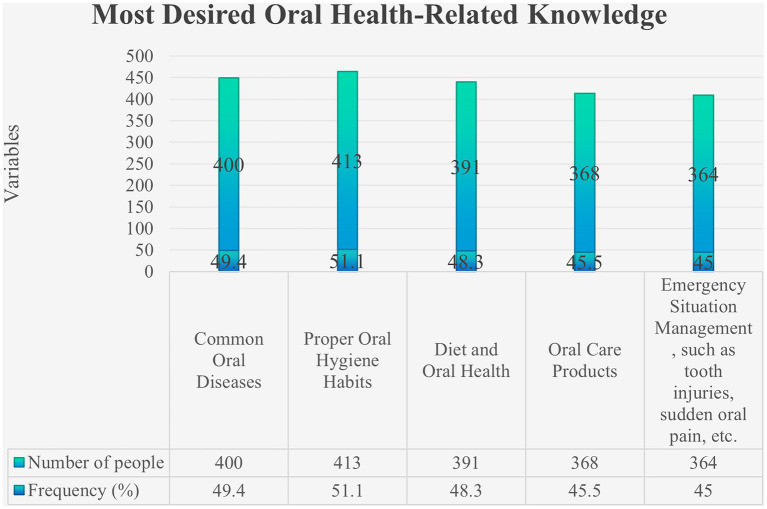
Most desired oral health-related knowledge.

**Figure 4:**
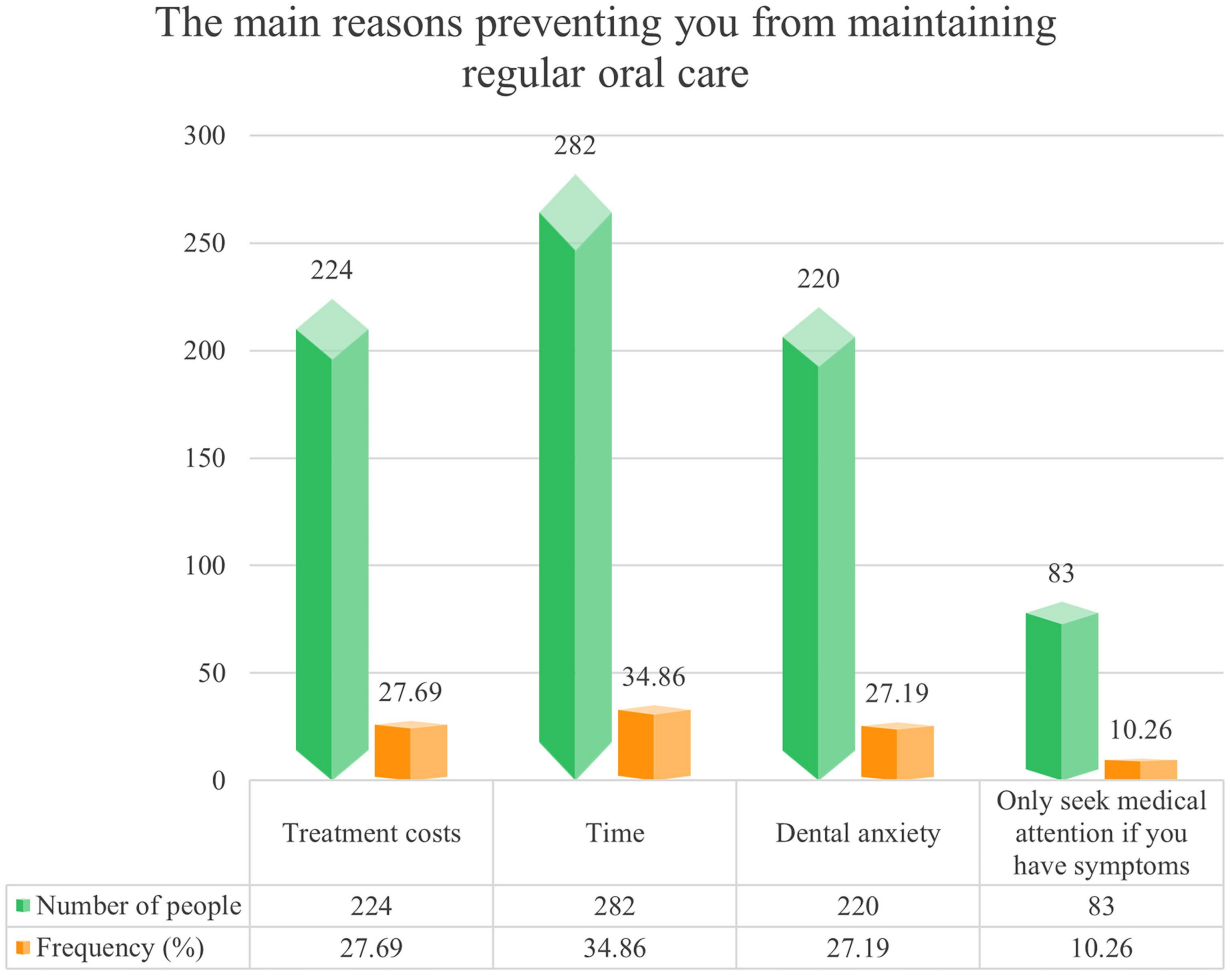
The main reasons preventing you from maintaining regular oral care.

Most participants reported obtaining oral health information from books, academic journals, and newspapers (Figure 2), and only 41.16% had received systematic oral health education. Regarding training needs, correct oral hygiene habits were the topic participants most wanted to learn about (Figure 3). In addition, 34.86% of participants reported that lack of time was a major barrier to regular oral health care (Figure 4).

### 3.7 Correlation analysis

Spearman correlation analysis showed that practice scores were positively correlated with attitude scores (*r* = 0.203, *p* < 0.01), and attitude scores were also positively correlated with knowledge scores (*r* = 0.143, *p* < 0.01). In contrast, knowledge scores were not significantly correlated with practice scores (*p* > 0.05) (Table 6).

**Table 6:** Spearman correlation analysis between knowledge, attitude, and practice scores.

Variable	Total behavior score	Total attitude score	Total knowledge score
Practice	1		
Attitude	0.203**	1	
Knowledge	0.014	0.143**	1

**p < 0.01.

### 3.8 Ordinal logistic regression analysis

Overall KAP score was used as the dependent variable. Independent variables included sex, age, education, years of work, professional title, birthplace, marital status, profession, prior oral health education, and prompt care-seeking for oral discomfort. Variable coding is shown in Table 7.

**Table 7:** Coding of independent variables.

Independent variable	Assignment specifications
Sex	Male = 0; Female = 1
Age(years)	18–34 = 1; 35–44 = 2; 45–54 = 3; ≥55 = 4
Educational	Bachelor’s degree or below = 0; Master’s degree or above = 1
Years of work	1–5 = 1; 6–10 = 2; 11–15 = 3; ≥16 = 4
Professional title	Junior = 1; Intermediate = 2; Senior = 3
Birthplace	Rural = 0; Urban = 1
Marital status	Unmarried = 0; Married = 1
Profession	Clinical Medicine = 1; Nursing = 2; Other medical disciplines = 3
Prior oral health education	No = 0; Yes = 1
Seek care promptly when experiencing oral discomfort	No = 0; Yes = 1

The ordinal logistic regression model fit the data well (model chi-square = 161.436, *df* = 16, *p* < 0.001). The goodness-of-fit test indicated acceptable fit (deviance chi-square = 662.612, *p* = 0.055). The test of parallel lines was non-significant (*p* = 0.076), supporting the proportional odds assumption. Age, education, marital status, and prior oral health education were significantly associated with overall KAP level. Participants with higher education (master’s degree or above) and those who had received oral health education were more likely to have higher KAP levels, whereas younger participants (18–34 years) and married participants tended to score lower. Detailed results are shown in Table 8.

**Table 8:** Ordered logistic regression analysis results.

Variable	*β*	OR	95% CI	p-value
Age (years)				
18–34	0.445	1.56	0.61–3.85	0.353
35–44	0.357	1.43	0.49–4.02	0.292
45–54	0.714	2.04	1.14–4.92	0.001**
≥55	Reference	1	—	—
Education (master’s or above vs. bachelor’s or below)	1.192	3.29	1.37–7.86	0.007**
Marital status (married vs. Unmarried)	−1.438	0.24	0.08–0.58	0.025*
Prior oral health education (Yes vs. No)	1.287	3.62	1.61–8.16	<0.001**

*p < 0.05.

## 4 Discussion

This cross-sectional survey of 809 NDHPs in South China systematically assessed their oral health knowledge, attitudes, and practices and identified key associated factors. Overall, NDHPs showed positive attitudes toward oral health but had limited knowledge and suboptimal implementation of healthy behaviors. These findings suggest that knowledge may be a major bottleneck in the pathway from awareness to practice.

Overall KAP levels were mainly associated with age, educational attainment, marital status, and prior oral health education. Older participants tended to have greater oral health awareness, whereas married participants were more likely to have lower KAP levels. One possible explanation is that married adults often face greater family responsibilities, caregiving demands, and work–family conflicts, which may reduce the time, energy, and attention available for self-care and preventive health behaviors (25–28). In this context, even when oral health awareness is present, the translation of knowledge and attitudes into sustained healthy practices may still be hindered by competing daily demands. This interpretation is broadly consistent with previous studies showing that health behaviors are shaped not only by individual knowledge, but also by broader social support, family context, and lifestyle constraints (25, 27, 28). Therefore, the association between marital status and lower KAP scores observed in this study may reflect the combined influence of social-role burden and contextual barriers rather than marital status itself as an isolated determinant. Moreover, this finding may also have been influenced by unmeasured confounding factors that were not captured in the present study and should therefore be interpreted with caution. In addition, higher education attainment and prior systematic oral health education were associated with higher overall KAP levels (29). Based on the Spearman correlation analysis and ordinal logistic regression, our findings suggest that positive attitudes may play an important intermediary role in the translation of knowledge into behavior, which is broadly consistent with the IKAP pathway (13, 30). Overall, these results provide empirical support for understanding the KAP characteristics of NDHPs and may help inform the development of targeted oral health promotion strategies.

Several features strengthen the credibility of our findings. First, the sample size was large and included NDHPs from multiple disciplines, improving representativeness. Second, we applied complementary analyses-between-group comparisons, correlation analyses, and ordinal logistic regression-to triangulate findings from descriptive patterns to associations and determinants. Moreover, the questionnaire and analytic strategy were aligned with the IKAP framework, enhancing interpretability (13). Finally, the questionnaire was refined based on prior evidence and NDHPs’ professional characteristics, supporting the scientific rigor and stability of the collected data.

A key finding of this study was low knowledge but relatively high attitudes, suggesting that positive attitudes may not arise solely from professional oral health knowledge but may also reflect broader professional health awareness. We also observed profession-specific strengths in particular behaviors: participants from other medical disciplines performed better on brushing duration, clinicians reported relatively better control of high-sugar intake, and nurses reported higher use of fluoridated toothpaste and mouthwash. These differences suggest that oral health behaviors are shaped not only by general knowledge but also by professional training emphases, everyday clinical contexts, and role expectations (31). Importantly, “lack of time” was commonly reported as a major barrier, indicating that workload and contextual constraints may directly limit behavior implementation, potentially outweighing knowledge itself. Overall, oral health practices among NDHPs appear to be jointly influenced by knowledge, health consciousness, professional role, and environmental factors.

Consistent with prior studies (32, 33), our findings further confirm that healthcare professionals may hold positive attitudes toward oral health but still exhibit significant gaps in knowledge and practice. Importantly, in this NDHPs population we observed that knowledge was not significantly associated with practice, whereas attitude showed a stable positive correlation with behavior, suggesting that attitude may play an important role in the process through which knowledge is translated into behavior. This finding is highly consistent with the IKAP framework, which proposes that information and knowledge are internalized into attitudes before being translated into practice, and it further supports the applicability and explanatory value of IKAP in multidisciplinary healthcare populations. However, because this study was cross-sectional, these associations should not be interpreted as evidence of mediation or causal pathways. Future longitudinal or interventional studies are still needed to clarify the temporal sequence and potential causal relationships among knowledge, attitudes, and behaviors.

As one of the few surveys focusing on NDHPs in South China, this study systematically compared differences in KAP across three professional groups and revealed the association between occupational background and specific behavioral patterns. By comprehensively examining the combined effects of age, educational attainment, marital status, and oral health education experience on KAP levels, this study further enriches current understanding of the factors associated with oral health-related behaviors among healthcare professionals (28, 34–36). The findings not only highlight the importance of systematic education and health promotion in improving behaviors, but also suggest that family and work burdens may constitute structural barriers to behavioral implementation, thereby providing a useful basis for developing more precise oral health education programs for NDHPs.

However, although 41.16% of participants reported having received systematic oral health education, the overall knowledge correct rate remained low (25.94%). Participants with prior oral health education were more likely to have higher overall KAP levels and had significantly higher knowledge scores than those without such education; nevertheless, the median knowledge score in the educated group was still only 3, indicating that the overall knowledge level remained low. This suggests that prior education may confer some benefit, but its effect at the population level remains limited. One possible explanation is that the education received by NDHPs may still have been limited in depth, continuity, standardization, or practical reinforcement. Another possibility is that some training programs place greater emphasis on general health awareness and preventive attitudes than on structured knowledge mastery. Previous studies have shown that oral health education can improve awareness and selected practice-related outcomes among non-dental healthcare professionals, but knowledge gaps often persist when oral health training is insufficiently integrated into non-dental curricula or lacks consistency (10, 34, 36). Therefore, future educational strategies should place greater emphasis on curriculum quality, repeated reinforcement, clinical applicability, and measurable learning outcomes to improve long-term knowledge retention and its translation into practice.

Finally, nurses showed slightly better performance on some knowledge and practice items, potentially reflecting nursing training that emphasizes holistic care, health education, and patient self-management, as well as nursing roles that involve substantial patient management and counseling (10, 32). However, these advantages did not translate into large overall differences, suggesting that nursing-related benefits may derive more from practical experience and adherence to workflows than from a systematic knowledge structure. This aligns with the broader concern that oral health content remains insufficient in medical education systems (10, 36). The aim of this study is not to advocate for systemic reform but to provide evidence to inform the development and implementation of oral health education and practice.

## 5 Limitations

This study has several limitations. First, as a cross-sectional survey, it can describe current KAP levels and associated factors but cannot establish causal relationships. Future longitudinal or interventional studies are needed to evaluate dynamic changes and causal pathways among knowledge, attitudes, and practices. Second, participants were recruited from South China; regional, cultural, and educational differences may limit generalizability. Future studies should include NDHPs from additional regions and from different levels of healthcare institutions. In addition, the distribution of marital status in our sample was highly imbalanced, with married participants accounting for the overwhelming majority. This imbalance may have affected the stability and precision of the regression estimate for marital status and therefore warrants cautious interpretation. Future studies should include more balanced samples or larger numbers of unmarried participants to further verify the robustness of this association. Third, the questionnaire focused primarily on basic knowledge, behaviors and attitudes and did not comprehensively assess constructs such as oral health literacy scales or self-efficacy, which may mediate translation from knowledge to practice. In addition, we did not further assess the content, duration, intensity, recency, or delivery format of prior oral health education, so the effectiveness of different educational models could not be directly evaluated. Oral health behaviors were also self-reported and may therefore have been affected by reporting bias and social desirability bias. Future research could incorporate more detailed educational exposure measures, validated literacy-related instruments, and objective behavioral indicators for a more comprehensive assessment.

## 6 Conclusion

NDHPs in South China generally hold positive attitudes toward oral health but have limited knowledge and suboptimal behavioral implementation, indicating substantial room for improvement in overall KAP levels. Educational attainment, prior training, and age were important determinants of overall KAP level, while marital status may constrain behavior implementation due to family and work burdens. These findings suggest that oral health content should be strengthened in undergraduate medical education and continuing professional development for NDHPs. Moreover, stratified and tailored oral health promotion measures should be developed for NDHPs subgroups with different professional roles and characteristics to enhance their contributions to patient oral health management.

## Data Availability

The raw data supporting the conclusions of this article will be made available by the authors, without undue reservation.
